# Efficacy and safety of almonertinib as adjuvant therapy in stage II-IIIA EGFR-mutant non-small cell lung cancer: a retrospective study

**DOI:** 10.3389/fmed.2026.1891244

**Published:** 2026-08-06

**Authors:** Tingting Cao, Ling Chen, Ying Li, Shuang Wu, Ying Liu, Wenjing Tian, Yiqing Zhang, Yuezhen An, Ming Wang, Zefen Lu, Qiutong Wang

**Affiliations:** 1 Department of Medical Technology, Cangzhou Medical College, Cangzhou, Hebei, China; 2 Department of Nursing, Cangzhou Medical College, Cangzhou, Hebei, China; 3 Department of Pharmacy, Cangzhou Medical College, Cangzhou, Hebei, China; 4 Department of Basic Medical Sciences, Cangzhou Medical College, Cangzhou, Hebei, China; 5 Department of Clinical Medicine, Cangzhou Medical College, Cangzhou, Hebei, China; 6 Department of Thoracoscopic Surgery, Cangzhou Central Hospital, Cangzhou, Hebei, China; 7 Department of Clinical Laboratory, Cangzhou Central Hospital, Cangzhou, Hebei, China; 8 Department of Oncology, Cangzhou Central Hospital, Cangzhou, Hebei, China; 9 Department of Oncology, Cangzhou People's Hospital (Cangzhou Medical College Affiliated Hospital), Cangzhou, Hebei, China

**Keywords:** adjuvant therapy, almonertinib, central nervous system, efficacy, EGFR mutation

## Abstract

**Objective:**

To compare the efficacy and safety of adjuvant almonertinib versus icotinib in patients with resected stage II-IIIA epidermal growth factor receptor (EGFR)-mutant non-small cell lung cancer (NSCLC).

**Methods:**

This retrospective study included 80 patients treated with adjuvant almonertinib or icotinib after complete resection. Disease-free survival (DFS) was the primary endpoint; secondary endpoints included overall survival (OS) and safety. Multivariate Cox regression was used to identify factors associated with DFS and OS.

**Results:**

Baseline characteristics were well balanced. After a median follow-up of 40.0 months, almonertinib demonstrated a significant DFS advantage (HR = 0.36, 95% CI: 0.16–0.82), with the median DFS not reached versus 29.0 months for icotinib (*p* = 0.0142). The 24-month DFS rates were 85.1 and 61.2%, respectively. Subgroup analyses showed consistent benefits in male patients, patients younger than 65 years, patients with stage IIIA disease and those with smoking history. OS also favored almonertinib (HR = 0.43, 95% CI: 0.19–0.94), with the median OS not reached versus 41.2 months (*p* = 0.0355). Recurrence occurred in 17.5% of the almonertinib group and 45.0% of the icotinib group (*p* = 0.0150). The 24-month central nervous system (CNS) metastasis-free survival rate reached 100% in the almonertinib group versus 96.6% in the icotinib group; however, the total number of CNS metastatic events was very small (*n* = 3), so this numerical difference should only be interpreted as an exploratory trend rather than definitive evidence of CNS protection. Safety profiles were comparable, although grade ≥3 TEAEs were less frequent with almonertinib (7.5% vs. 25.6%, *p* = 0.0367). No TEAEs or TRAEs leading to death were observed.

**Conclusion:**

In this retrospective cohort, adjuvant almonertinib was associated with significantly improved DFS and OS, recurrence prevention, and a more favorable safety profile compared with icotinib. A numerical trend of reduced CNS metastasis was observed, but limited CNS events preclude firm conclusions regarding CNS protective activity. A tentative early OS survival trend favoring almonertinib was observed, but OS data remain immature with limited death events; long-term follow-up is needed to verify this potential survival benefit.

## Introduction

Lung cancer remains the leading cause of cancer-related mortality worldwide, with non-small-cell lung cancer (NSCLC) accounting for approximately 85% of all cases. Although surgical resection is potentially curative for early-stage disease, postoperative recurrence remains a major challenge. Large meta-analyses indicate that 45% of stage IB and up to 76% of stage III patients eventually relapse, even after complete resection and standard adjuvant platinum-based chemotherapy (1). Moreover, as disease stage increases, the risk of local recurrence, distant metastasis, and particularly central nervous system (CNS) involvement rises substantially, severely compromising quality of life. For patients with stage III NSCLC, the 5-year overall survival (OS) remains only 20–30% (2), underscoring a significant unmet clinical need.

Pooled genomic epidemiology encompassing 115, 815 NSCLC patients worldwide shows a global epidermal growth factor receptor (EGFR)-sensitizing mutation frequency of 32.3% (95% CI: 30.9–33.7%) (3), ranging from 38.4% (95% CI: 36.5 to 40.3%) in China to 14.1% (95% CI: 12.7 to 15.5%) in Europe, with most commonly exon 19 deletions (19Del) and exon 21L858R substitutions (21L858R). These tumors exhibit oncogene addiction to EGFR signaling, providing a strong biological rationale for targeted inhibition. The introduction of EGFR tyrosine kinase inhibitors (EGFR-TKIs) has transformed the treatment landscape of advanced EGFR-mutant NSCLC and prompted exploration of their use in the adjuvant setting (4). Among first-generation EGFR-TKIs, icotinib has become one of the most widely used agents in China due to its favorable toxicity profile, affordability, and demonstrated efficacy in both metastatic and postoperative settings (5). Several randomized studies have shown that icotinib improves disease-free survival (DFS) compared with chemotherapy (6, 7); however, its clinical benefit is limited by suboptimal CNS penetration and the inevitability of resistance, particularly the emergence of the T790M mutation (8).

The advent of third-generation EGFR-TKIs has substantially reshaped the therapeutic paradigm. Osimertinib, a potent irreversible inhibitor with strong activity against both sensitizing and T790M mutations, demonstrated a 77% reduction in disease recurrence or death (HR = 0.23) in completely resected stage II-IIIA EGFR-mutant NSCLC (9), as shown in the landmark ADAURA trial. Notably, osimertinib also significantly reduces CNS metastasis (10), addressing one of the most clinically problematic relapse patterns in early-stage EGFR-mutant NSCLC. Despite these advances, Osimertinib’s high cost and limited accessibility restrict its widespread use, especially in resource-constrained healthcare systems.

Almonertinib (also known as Aumolertinib or HS-10296), a recently developed third-generation EGFR-TKI with potent and selective inhibition of sensitizing and T790M resistance mutations, provides a promising therapeutic option due to its enhanced CNS penetration and clinical availability (11). The landmark multicenter randomized phase III ARTS trial established prospective evidence supporting adjuvant almonertinib; compared with placebo, adjuvant almonertinib significantly prolonged disease-free survival (DFS) in patients with completely resected stage II-IIIB EGFR-mutated lung adenocarcinoma. Corresponding subgroup analyses further validated its durable intracranial tumor control capacity and well-tolerated adverse event spectrum during long-term follow-up (12). Meanwhile, the EVIDENCE phase III trial verified adjuvant icotinib’s DFS benefit relative to chemotherapy (6). Direct head-to-head randomized data comparing adjuvant almonertinib and icotinib are still absent, leaving the relative efficacy and safety of these two oral adjuvant TKIs unclarified in real-world clinical practice. While almonertinib has demonstrated clinically meaningful antitumor activity in advanced EGFR-mutant NSCLC, its comparative efficacy and safety versus first-generation EGFR-TKIs in the postoperative adjuvant setting remain largely unexplored.

Therefore, this study aims to assess the efficacy and safety of adjuvant almonertinib versus icotinib in patients with completely resected stage II-IIIA EGFR-mutant lung adenocarcinoma. Existing prospective phase III data only provide controlled trial evidence under strict standardized inclusion and exclusion criteria, while large-scale real-world comparative data reflecting routine clinical practice remain scarce. This work stands out by delivering long-term follow-up DFS and safety outcomes from unselected real-world cohorts, which complements rigid trial-derived data and offers more generalizable clinical references for domestic postoperative targeted treatment decisions. By filling this unaddressed evidence gap, the present retrospective analysis provides essential real-world insights to guide optimization of adjuvant targeted therapy in EGFR-mutant NSCLC.

## Materials and methods

### Study population

This retrospective study included patients diagnosed with NSCLC and treated at Cangzhou Central Hospital and Cangzhou People’s Hospital between April 30, 2021, and March 31, 2023. Eligible patients were required to be ≥18 years old, have pathologically confirmed stage II-IIIA lung adenocarcinoma according to the 8th edition of the AJCC TNM classification, and harbor at least one measurable lesion with a sensitizing EGFR mutation. All patients had completed R0 pulmonary resection and subsequently received adjuvant therapy with either almonertinib or icotinib, with no prior exposure to icotinib before the initiation of almonertinib. Additional inclusion criteria comprised an Eastern Cooperative Oncology Group performance status (ECOG-PS) of 0–1 and the absence of distant metastasis confirmed by chest computed tomography (CT), brain CT, and magnetic resonance imaging (MRI). Patients were excluded if they had a history of other malignant tumors; received postoperative first- or second-generation EGFR-TKIs, radiotherapy, or other systemic anticancer therapies, radiotherapy, or other systemic anticancer therapies; discontinued targeted therapy within less than 1 month; or had incomplete clinical, follow-up, or revisit data. Notably, all enrolled patients received targeted adjuvant monotherapy only, and none of the subjects underwent platinum-based or any other postoperative adjuvant chemotherapy, eliminating adjuvant chemotherapy as a confounding prognostic factor. This dual-center retrospective observational study lacked a predefined prospective trial design; thus, no formal *a priori* sample size calculation was conducted before data collection. The final sample size was restricted by the limited number of patients meeting the unified adjuvant TKI inclusion criteria across the two participating hospitals within the study time window.

### EGFR mutation testing

All EGFR mutation detection was performed using formalin-fixed paraffin-embedded (FFPE) surgical tumor specimens collected from two study hospitals. EGFR exons 18–21 were detected using clinical-grade commercial NGS panels on Illumina sequencing platforms. Batch-based laboratory quality controls including positive and negative reference samples were applied in each run to guarantee reliable variant detection. Only classic sensitizing EGFR alterations (exon 19 deletion, exon 21 L858R) were defined as eligible mutations for enrollment; patients with T790M, exon 20 insertions or rare compound mutations were excluded.

### Treatment

Patients were non-randomly assigned to almonertinib or icotinib group according to comprehensive clinical decision-making by attending physicians, including patients’ financial affordability, expected treatment tolerance, disease stage and EGFR mutation subtype. Patients in the almonertinib group received almonertinib monotherapy at a dose of 110 mg administered orally once daily, which was continued for up to 2 years or until disease recurrence or the occurrence of unacceptable toxicity. In contrast, patients in the icotinib group were treated with icotinib at a standard dose of 125 mg administered orally three times daily, in accordance with routine clinical practice.

### Endpoints

The primary endpoint was DFS, defined as the interval from R0 resection to the first radiologically or pathologically confirmed recurrence of disease. Secondary endpoints included the overall survival (OS), CNS metastasis-free survival, the incidence of treatment-emergent adverse events (TEAEs) and treatment-related adverse events (TRAEs). Adverse events occurring during treatment were recorded and graded according to the National Cancer Institute Common Terminology Criteria for Adverse Events (CTCAE), version 5.0, with grades III-IV classified as severe.

### Follow-up

Patients were followed every 12 weeks during the first 2 years and every 24 weeks from the third through fifth years. Follow-up was terminated upon the diagnosis of disease recurrence, death, or loss to follow-up. Reporting of adverse events continued for 28 days after discontinuation of almonertinib or icotinib. The final follow-up date for this study was April 30, 2025.

### Recurrence assessment criteria

Disease recurrence was comprehensively judged by combined radiological and pathological evidence. Radiological suspicion of recurrence on chest/abdominal CT, brain MRI or bone scan required follow-up imaging to confirm progressive lesions; tissue biopsy or cytology was adopted as the gold standard to verify recurrence when feasible. Lymph node enlargement, new pulmonary nodules, distant organ lesions or CNS lesions meeting radiological progression thresholds were recorded as recurrence events.

### Statistical analysis

Statistical analyses were performed using GraphPad Prism (version 10.1). Categorical variables were summarized as frequencies and percentages. Differences between groups were compared using the chi-square test or Fisher’s exact test, as appropriate. Kaplan–Meier survival curves were generated to estimate DFS, and differences between treatment groups were assessed using the log-rank test. Between the two treatment groups. Strict eligibility screening was implemented prior to data extraction; all enrolled patients possessed complete baseline, survival follow-up and adverse event datasets with no missing values. No missing data processing methods (e.g., multiple imputation, complete case analysis) were applied in this study. Covariates for multivariate Cox regression were selected based on dual criteria: (1) clinicopathological variables with established prognostic significance for resected NSCLC; (2) variables with *p* < 0.05 in univariate Cox analysis. Schoenfeld residual test was performed to verify the proportional hazards (PH) assumption; all included covariates met the PH assumption without time-dependent interaction effects. For multivariate Cox proportional hazards regression, only variables with clinical relevance and significant univariate prognostic associations were included stepwise to avoid redundant covariates; the final models only retained two independent predictors (disease stage and visceral pleural involvement). A total of 25 DFS events and 20 OS events were recorded, satisfying the standard “10 events per covariate” rule and ruling out model overfitting. Multivariable Cox proportional hazards regression was performed to identify independent prognostic factors for DFS and OS. All baseline clinicopathological features were well balanced at enrollment to minimize selection bias, and multivariate Cox models were applied to further adjust key prognostic confounders instead of propensity score matching (PSM) or inverse probability of treatment weighting (IPTW), since PSM/IPTW would substantially reduce the limited sample size (*n* = 80) and compromise statistical power. All stratified subgroup survival analyses were pre-specified exploratory analyses only. Given the small overall sample size and limited number of DFS/OS events within each subgroup, subgroup results cannot be interpreted as definitive treatment effects and are only used to generate hypothesis for further validation. All statistical tests were two-sided, and a *p-*value <0.05 was considered statistically significant.

## Results

A total of 80 patients were included in the analysis, with 40 in the almonertinib group and 40 in the icotinib group. Baseline demographic and clinical characteristics were well balanced between the two groups, with no statistically significant differences observed (*p* > 0.05). The characteristics of the two groups are summarized in Table 1. The proportions of male and female patients, age distribution, smoking history, EGFR mutation type, ECOG performance status (ECOG-PS), and disease stage were comparable between the two treatment groups.

**Table 1:** Baseline characteristics of the two treatment groups.

Variable	Almonertinib (*n* = 40)	Icotinib (*n* = 40)	*p* value
Sex, *n* (%)			0.822^1^
Male	18 (45.0%)	17 (42.5%)	
Female	22 (55.0%)	23 (57.5%)	
Age, *n* (%)			0.822^1^
<65 years	22 (55.0%)	23 (57.5%)	
≥65 years	18 (45.0%)	17 (42.5%)	
Smoking history, *n* (%)			0.592^1^
Yes	30 (75.0%)	32 (80.0%)	
No	10 (25.0%)	8 (20.0%)	
EGFR mutation type, *n* (%)			0.653^1^
19Del	23 (57.5%)	21 (52.5%)	
21L858R	17 (42.5%)	19 (47.5%)	
ECOG-PS, *n* (%)			0.496^1^
0	18 (45.0%)	15 (37.5%)	
1	22 (55.0%)	25 (62.5%)	
Disease stage, *n* (%)			0.799^1^
Stage II	11 (27.5%)	10 (25.0%)	
Stage IIIA	29 (72.5%)	30 (75.0%)	
Tumor diameter, *n* (%)			0.635^1^
<45 mm	21 (52.5%)	23 (57.5%)	
≥45 mm	19 (47.5%)	17 (42.5%)	
Type of anatomic resection, *n* (%)			0.615^2^
Lobectomy	39 (97.5%)	37 (92.5%)	
Bilobectomy	1 (2.5%)	3 (7.5%)	
Visceral pleural involvement, *n* (%)			0.499^2^
No	24 (60.0%)	21 (52.5%)	
Yes	16 (40.0%)	19 (47.5%)	
Vascular invasion			0.633^1^
No	28 (70.0%)	26 (65.0%)	
Yes	12 (30.0%)	14 (35.0%)	
Mediastinal lymph node metastasis, *n* (%)			0.818^1^
No	24 (60.0%)	25 (62.5%)	
Yes	16 (40.0%)	15 (37.5%)	

1 Pearson’s Chi-squared test.

2 Fisher’s exact test.

Zero patients in both groups received postoperative adjuvant chemotherapy (Almonertinib: 0/40, Icotinib: 0/40).

**Figure 1:**
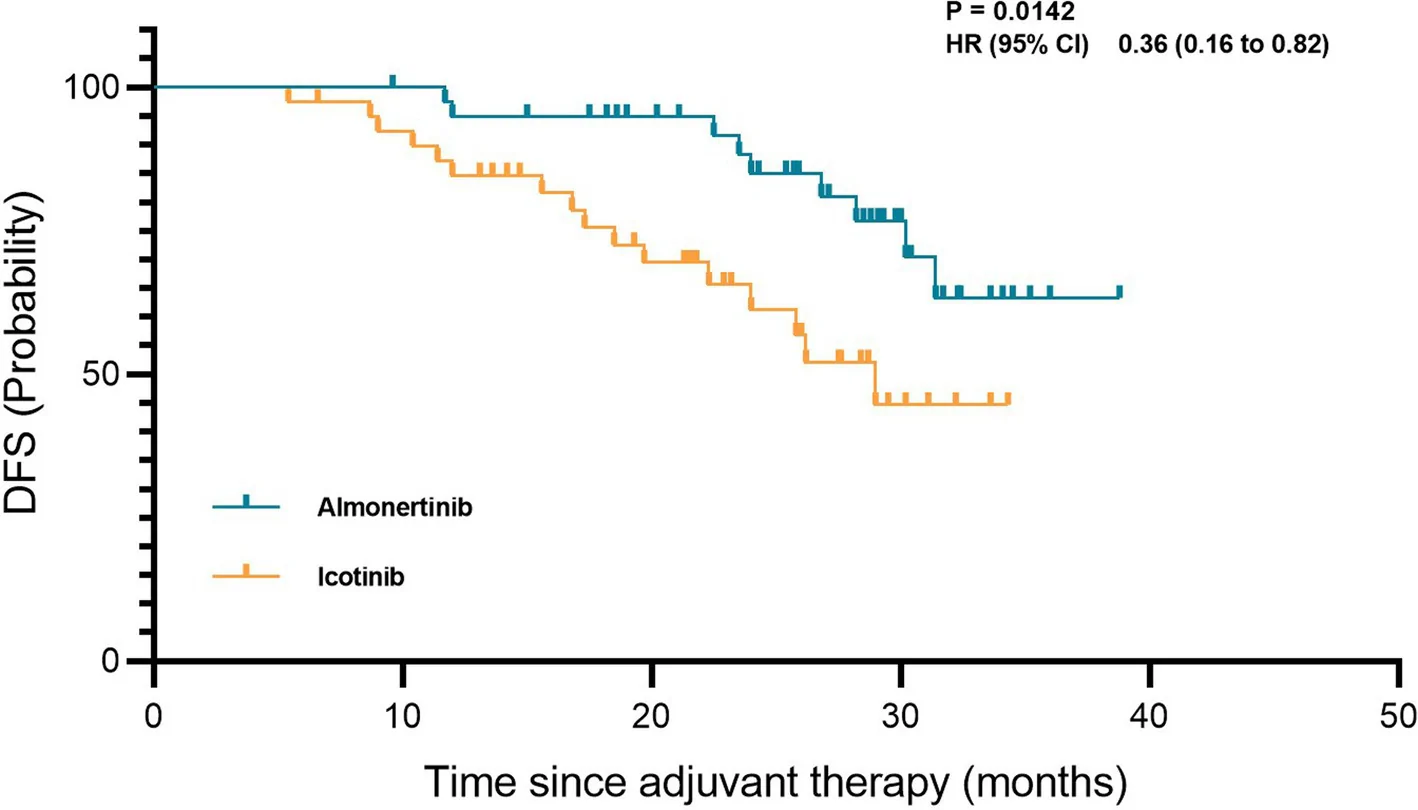
Kaplan–Meier curves of disease-free survival (DFS) in the two treatment groups.

The median follow-up duration was 40.0 months (95% CI: 37.7–42.3) for the entire cohort, with 39.0 months (95% CI: 36.6–41.4) in the almonertinib group and 40.0 months (95% CI: 37.0–42.3) in the icotinib group. In the almonertinib group, 9 DFS events occurred (22.5% maturity) and 16 events occurred in the icotinib group (40.0% maturity); overall DFS maturity was 31.3%. The DFS HR was 0.36 (95% CI: 0.16–0.82). The median DFS was not reached (NR) in the almonertinib group, compared to 29.0 months in the icotinib group (*p* = 0.0142). The 2-year DFS rate was significantly higher in the almonertinib group (85.1%) compared to the icotinib group (61.2%), as shown in Figure 1.

**Figure 2:**
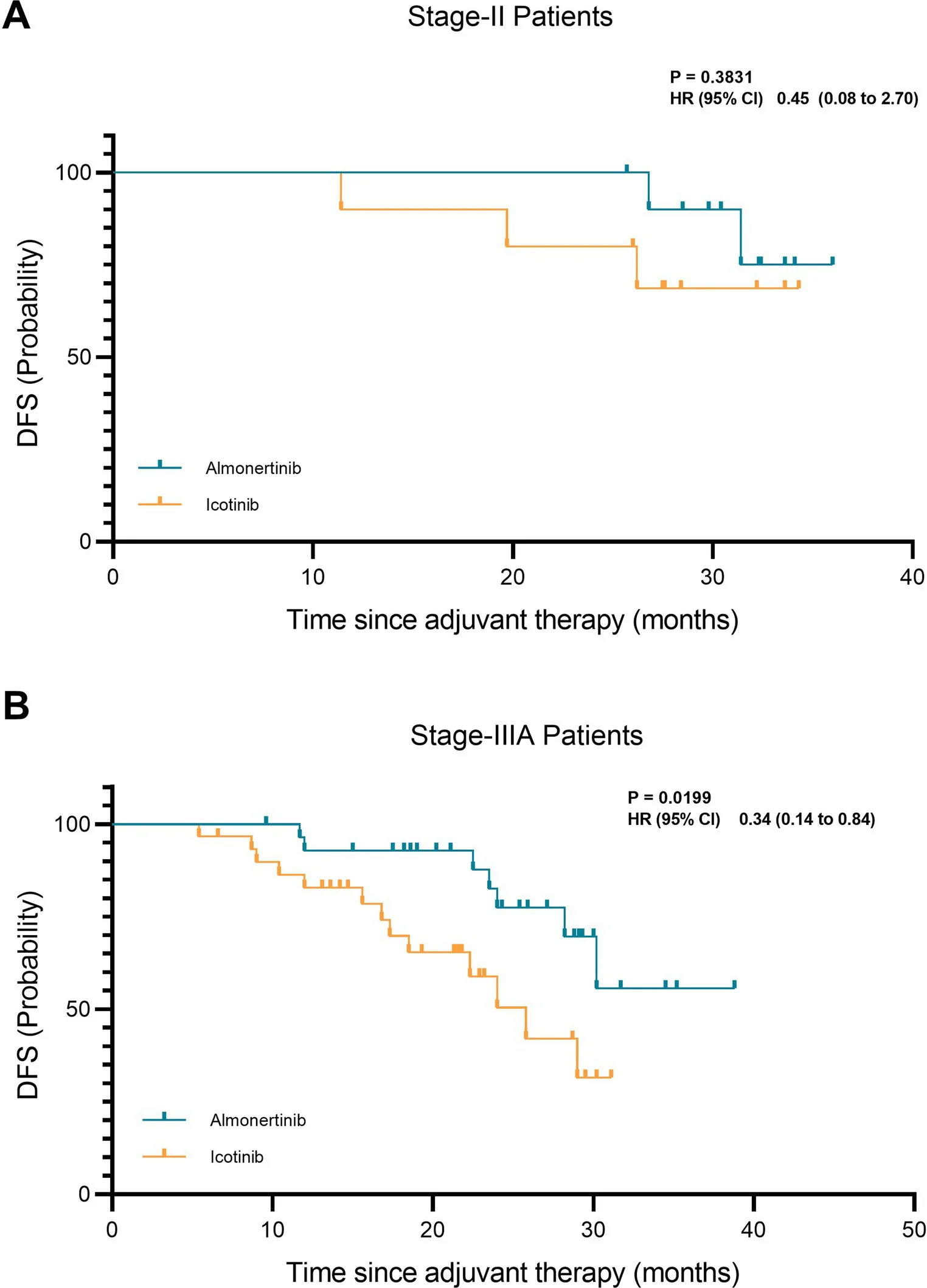
Kaplan–Meier curves of disease-free survival (DFS) stratified by disease stage. Subgroup analyses are exploratory only due to limited sample size and sparse events in each stratum. **(A)** DFS curves for stageII patients treated with almonertinib and icotinib. **(B)** DFS curves for stageIIIA patients treated with almonertinib and icotinib.

Among stage II patients, the mDFS was not reached in either the almonertinib or icotinib group (*p* = 0.3831), with a DFS HR of 0.41 (95% CI: 0.16–1.58). The 2-year DFS rates for stage II patients were 100% in the almonertinib group and 80.0% in the icotinib group. In stage IIIA patients, the mDFS was not reached with almonertinib versus 25.8 months with icotinib (*p* = 0.0199), with corresponding 2-year DFS rates of 77.4 and 50.5%, respectively, as shown in Figure 2; the DFS HR was 0.34 (95% CI: 0.14–0.84).

**Figure 3:**
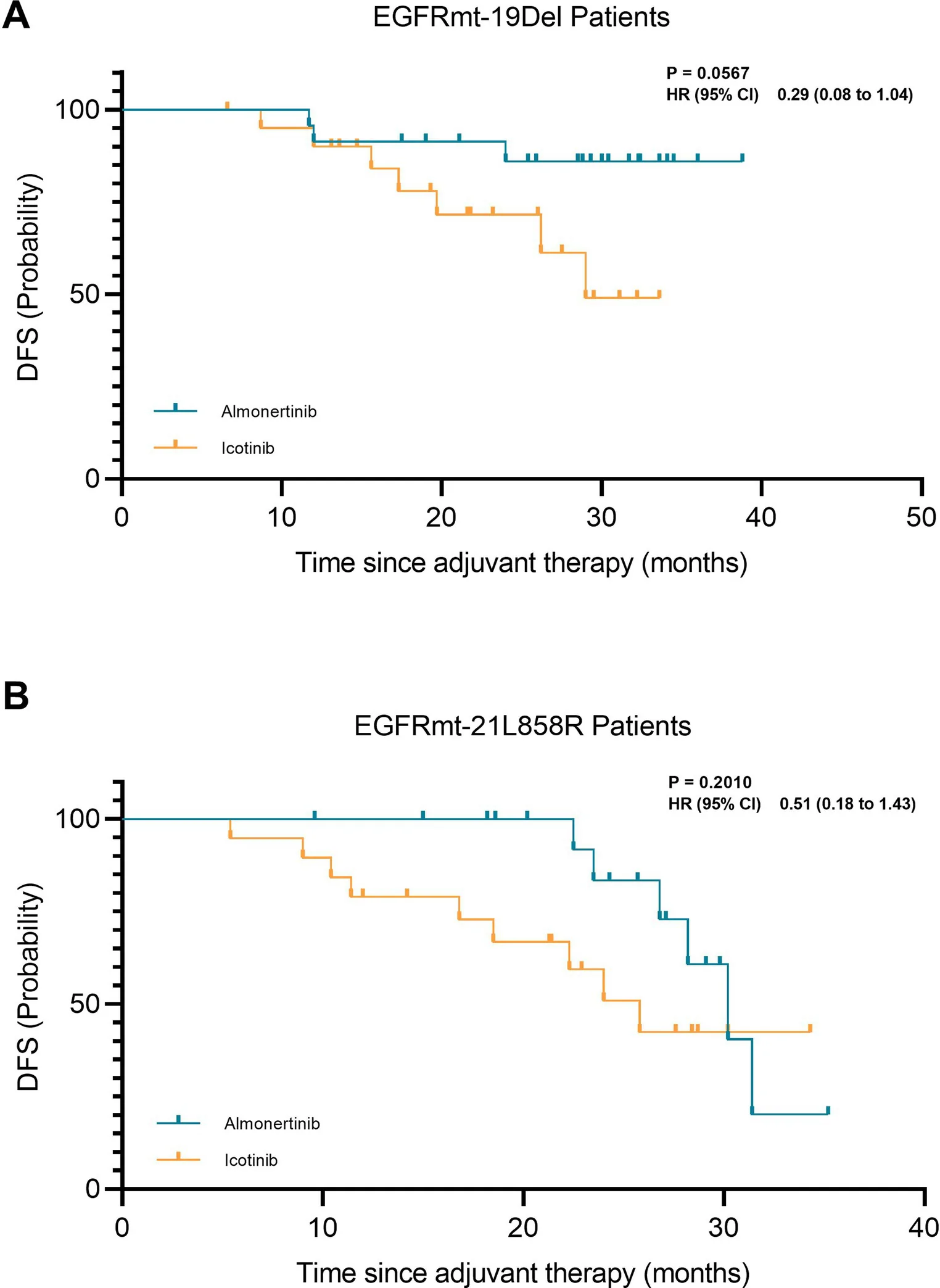
**Kaplan–Meier curves of disease-free survival (DFS) stratified by EGFR mutation subtype. Subgroup analyses are exploratory only due to limited sample size and sparse events in each stratum. (A)** DFS curves for 19Del mutation patients treated with almonertinib and icotinib. **(B)** DFS curves for 21L858R mutation patients treated with almonertinib and icotinib.

In patients with the 19Del mutation, the mDFS was not reached in the almonertinib group versus 29.0 months with icotinib (*p* = 0.0567), with a DFS HR of 0.29 (95% CI: 0.08–1.04). The 2-year DFS rate was 85.9% in the almonertinib group compared to 71.5% in the icotinib group. In patients with the 21L858R mutation, the mDFS was 30.2 months for almonertinib and 25.8 months for icotinib (*p* = 0.2010), with a DFS HR of 0.51 (95% CI: 0.18–1.43). 2-year DFS rates of 83.3 and 50.9%, respectively, as shown in Figure 3.

**Figure 4:**
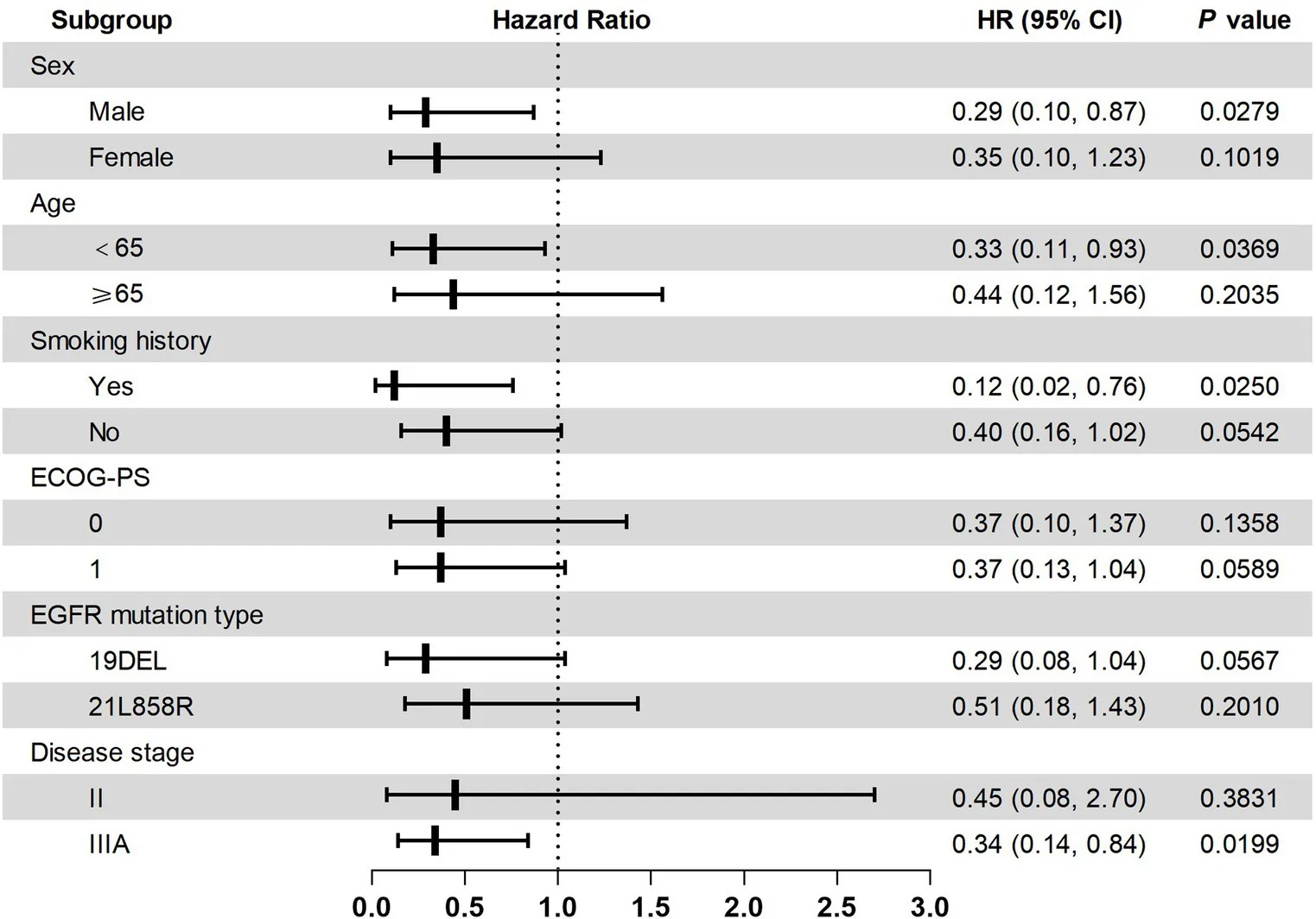
Forest plot of subgroup analyses for disease-free survival (DFS). All subgroup analyses are exploratory only; no definitive conclusion of treatment superiority can be drawn for any single subgroup due to limited sample and sparse events.

Exploratory subgroup analyses suggested tentative DFS signals favoring almonertinib in several strata, but these findings cannot support definitive conclusions of treatment superiority due to limited sample and sparse events per subgroup. Numerically better DFS was observed in male patients receiving almonertinib (HR = 0.29, 95% CI: 0.10–0.87; *p* = 0.0279), patients younger than 65 years (HR = 0.33, 95% CI: 0.11–0.93; *p* = 0.0369), and patients with smoking history (HR = 0.12, 95% CI: 0.02–0.76; *p* = 0.0250). These subgroup signals are hypothesis-generating only and require further validation in larger cohorts. Consistent with these findings, a significant DFS benefit was also observed among patients with stage IIIA disease (HR = 0.34, 95% CI: 0.14–0.84; *p* = 0.0199). In contrast, no statistically significant differences in DFS were detected between treatment groups within subgroups stratified by ECOG performance status or EGFR mutation type (all *p* > 0.05) (Figure 4).

**Figure 5:**
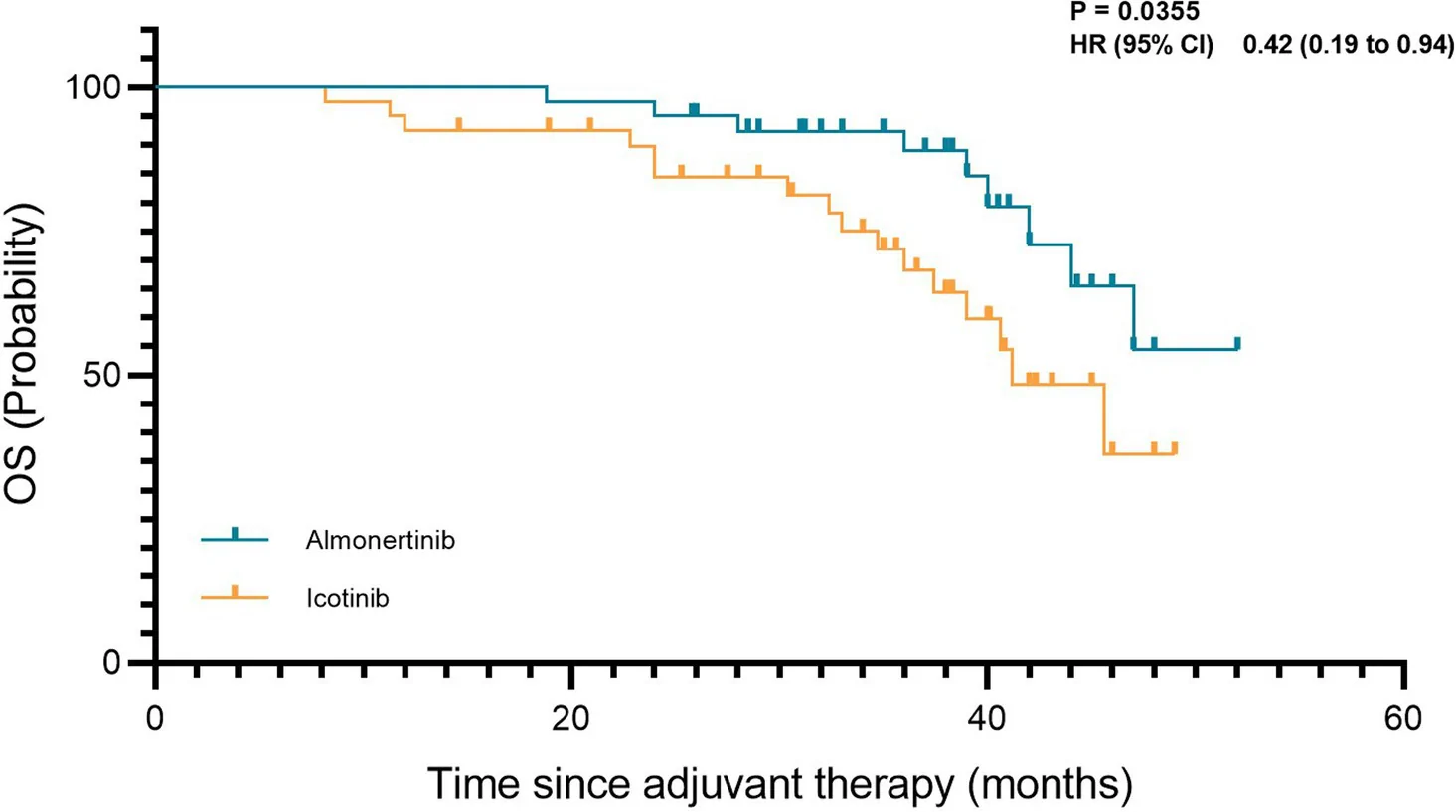
Kaplan–Meier curves of overall survival (OS) in the two treatment groups.

In the almonertinib group, 5 OS events occurred (12.5% maturity) and 15 events occurred in the icotinib group (37.5% maturity); overall OS maturity was 25%. Notably, OS data remain immature with a low total number of death events, so the OS survival difference between groups should be interpreted cautiously. The OS HR was 0.42 (95% CI: 0.19–0.94). The median OS was NR in the almonertinib group, compared to 41.2 months in the icotinib group (*p* = 0.0355) (Figure 5).

**Figure 6:**
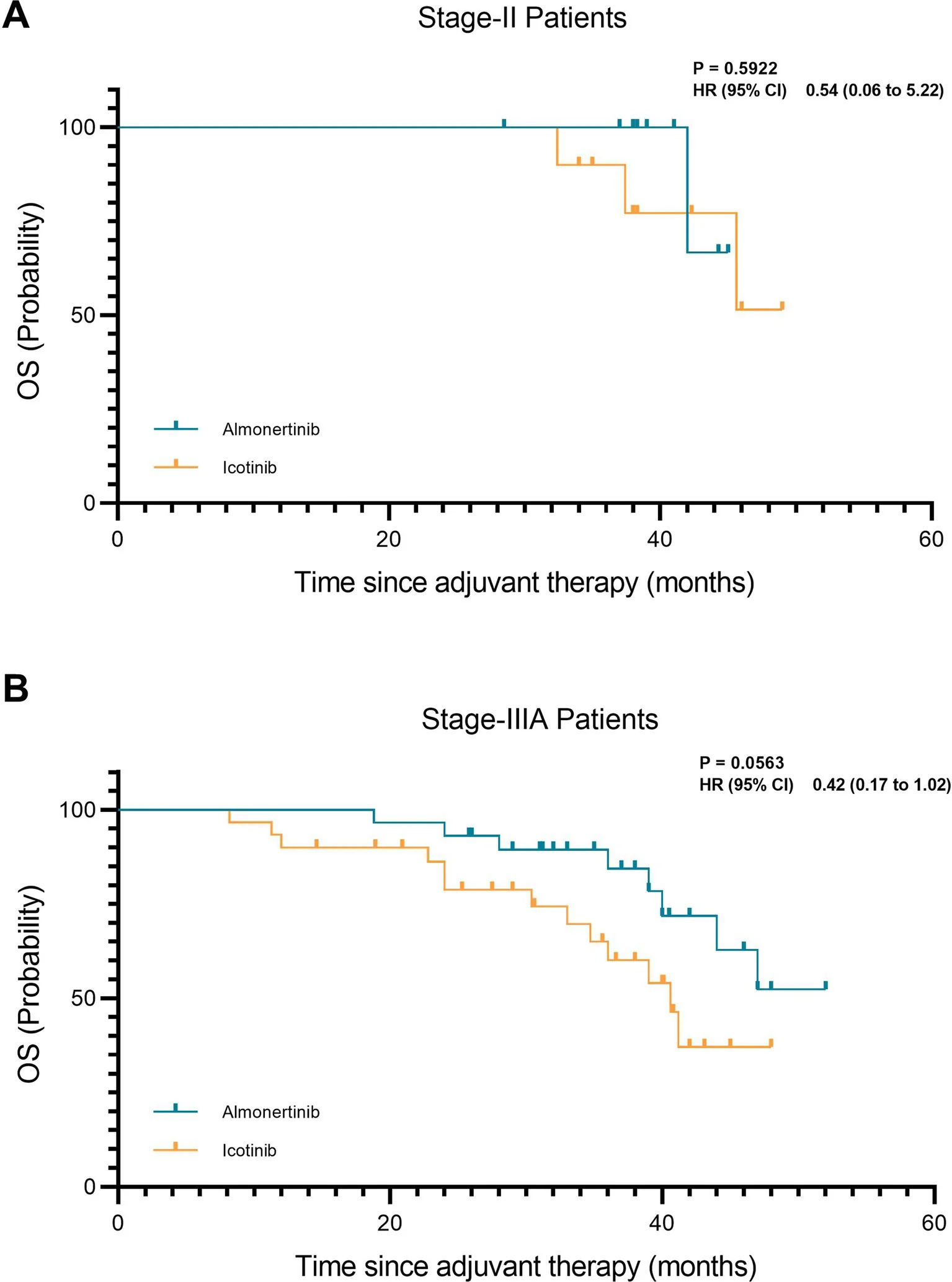
**Kaplan–Meier curves of overall survival (OS) stratified by disease stage. (A)** OS curves for stageII patients treated with almonertinib and icotinib. **(B)** OS curves for stageIIIA patients treated with almonertinib and icotinib.

Among patients with stage II disease, the mOS was not reached in either the almonertinib or icotinib group, with no statistically significant difference observed (HR = 0.54, 95% CI: 0.06–5.22; *p* = 0.5922). In the stage IIIA subgroup, mOS was not reached in the almonertinib group, whereas it was 40.6 months in the icotinib group (*p* = 0.0563). The corresponding 2-year OS rates were 93.1 and 78.8%, respectively (Figure 6). The OS HR favored almonertinib (HR = 0.42; 95% CI: 0.17–1.02).

**Figure 7:**
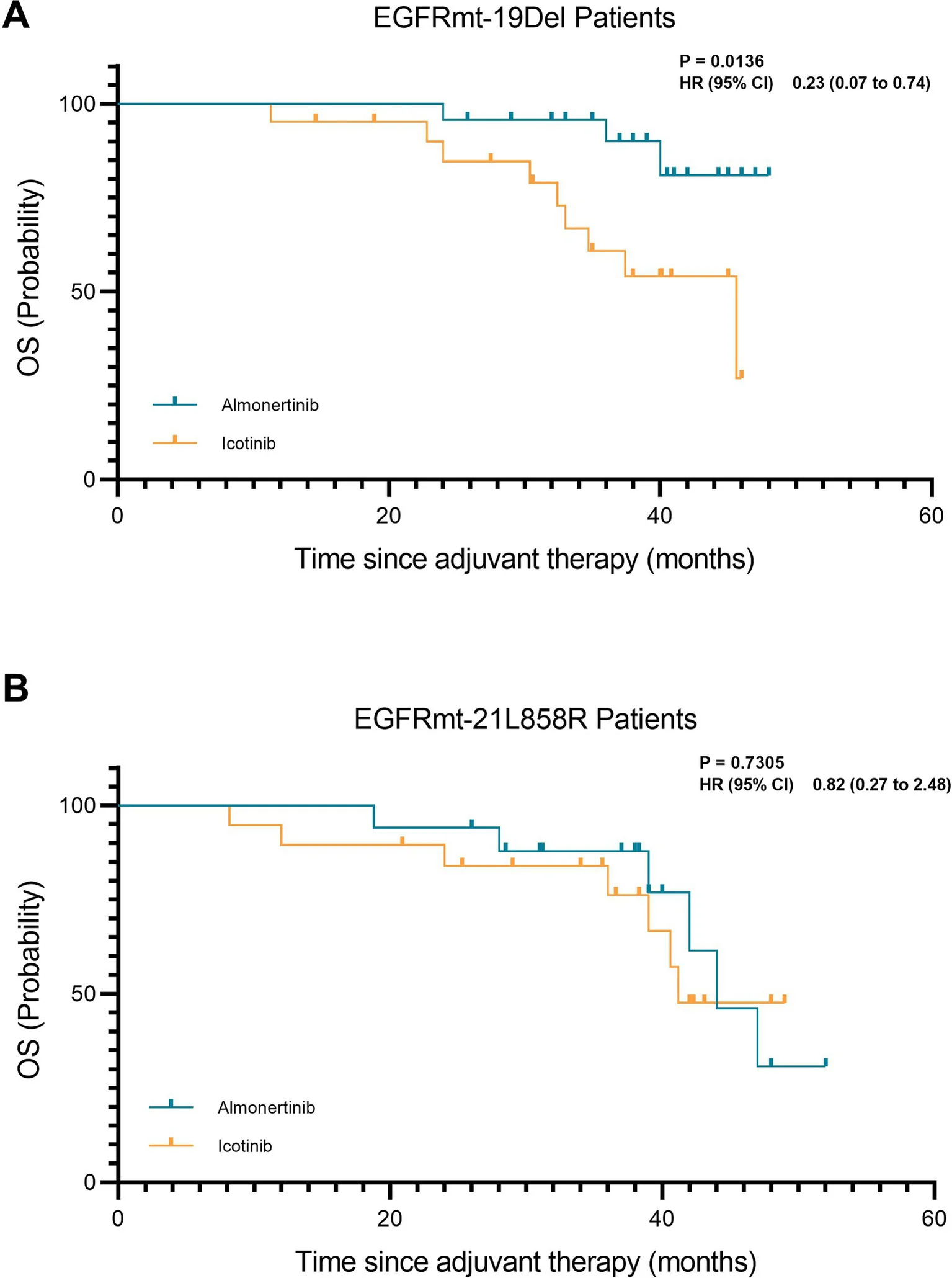
**Kaplan–Meier curves of overall survival (OS) stratified by EGFR mutation subtype. (A)** OS curves for 19Del mutation patients treated with almonertinib and icotinib. **(B)** OS curves for 21L858R mutation patients treated with almonertinib and icotinib.

Among patients harboring the 19Del mutation, mOS was not reached in the almonertinib group compared with 45.6 months in the icotinib group (*p* = 0.0136), corresponding to a significantly reduced risk of death (HR = 0.23, 95% CI: 0.07–0.74). The 2-year OS rate was 95.7% with almonertinib versus 84.7% with icotinib. Among patients with the 21L858R mutation, mOS was 44.0 months in the almonertinib group and 41.2 months in the icotinib group (*p* = 0.7305), with no significant difference in OS (HR = 0.82, 95% CI: 0.27–2.48). The corresponding 2-year OS rates were 94.1 and 83.9%, respectively (Figure 7).

Variables with known clinical relevance and potential prognostic value were included in the multivariate Cox model. Compared with stage II, patients with stage III disease had a significantly higher risk of recurrence (HR = 3.56, 95% CI: 1.09–11.63, *p* = 0.036). Similarly, visceral pleural involvement was independently associated with an increased risk of recurrence (HR = 2.88, 95% CI: 1.20–6.89, *p* = 0.018). For OS, disease stage remained the only significant factor, with stage IIIA patients experiencing a higher mortality risk than those with stage II (HR = 3.31, 95% CI: 1.01–10.87, *p* = 0.049). Other clinical and pathological characteristics, including sex, age, smoking history, EGFR mutation subtype, ECOG-PS, tumor size, vascular invasion, mediastinal nodal involvement, and type of pulmonary resection, were not independently associated with DFS or OS (*p* > 0.05) (Table 2).

**Table 2:** Multivariate analyses for factors associated with disease-free survival and overall survival.

Variable	Category	DFS		OS	
		Multivariate analysis		Multivariate analysis	
		HR (95% CI)	*p* value	HR (95% CI)	*p* value
Sex	Male vs. female	0.44 (0.19–1.05)	0.063	0.84 (0.35–2.03)	0.700
Age	<65 vs. ≥65	0.88 (0.37–2.09)	0.774	0.67 (0.27–1.65)	0.387
Smoking history	No vs. yes	1.03 (0.40–3.66)	0.958	0.42 (0.14–1.23)	0.112
EGFR mutation	19Del vs. 21L858R	1.61 (0.67–3.90)	0.292	1.41 (0.60–3.33)	0.437
ECOG PS	0 vs. 1	1.21 (0.51–2.85)	0.672	0.73 (0.31–1.69)	0.455
Disease stage	II vs. IIIA	3.56 (1.09–11.63)	0.036	3.31 (1.01–10.87)	0.049
Lung cancer resection type	Lobectomy vs. bilobectomy	0.38 (0.04–3.36)	0.386	0.71 (0.09–5.51)	0.743
Visceral pleural involvement	No vs. yes	2.88 (1.20–6.89)	0.018	1.40 (0.58–3.33)	0.453
Vascular invasion	No vs. yes	1.36 (0.57–3.25)	0.494	0.97 (0.40–2.38)	0.948
Mediastinal lymph node metastasis	No vs. yes	1.17 (0.47–2.87)	0.740	1.70 (0.69–4.20)	0.254
Tumor diameter	<45 mm vs. ≥45 mm	0.95 (0.38–2.37)	0.907	1.71 (0.72–4.07)	0.228

A total of 25 patients (31.3%) experienced disease recurrence or distant metastasis, with 7 (17.5%) in the almonertinib group and 18 (45.0%) in the icotinib group (*p* = 0.0150). The most common sites of recurrence were lymph nodes in both almonertinib group (5.0%) and icotinib group (10.0%) (Table 3).

**Table 3:** Patterns of disease recurrence and first sites of relapse.

Recurrence type/site	Almonertinib (*n* = 40)	Icotinib (*n* = 40)
Total recurrences	7 (17.5%)	18 (45.0%)
Local or regional recurrence only	5 (12.5%)	10 (25.0%)
Distant recurrence only	2 (5.00%)	5 (22.5%)
Local/regional + distant recurrence	0	3 (7.50%)
Death	0	1 (2.50%)
First sites of recurrence		
Lung	0	3 (7.50%)
Lymph nodes	2 (5.00%)	4 (10.0%)
Bone	1 (2.50%)	2 (5.00%)
Pleura	0	1 (2.50%)
Liver	0	1 (2.50%)
CNS only	1 (2.50%)	2 (5.00%)

**Figure 8:**
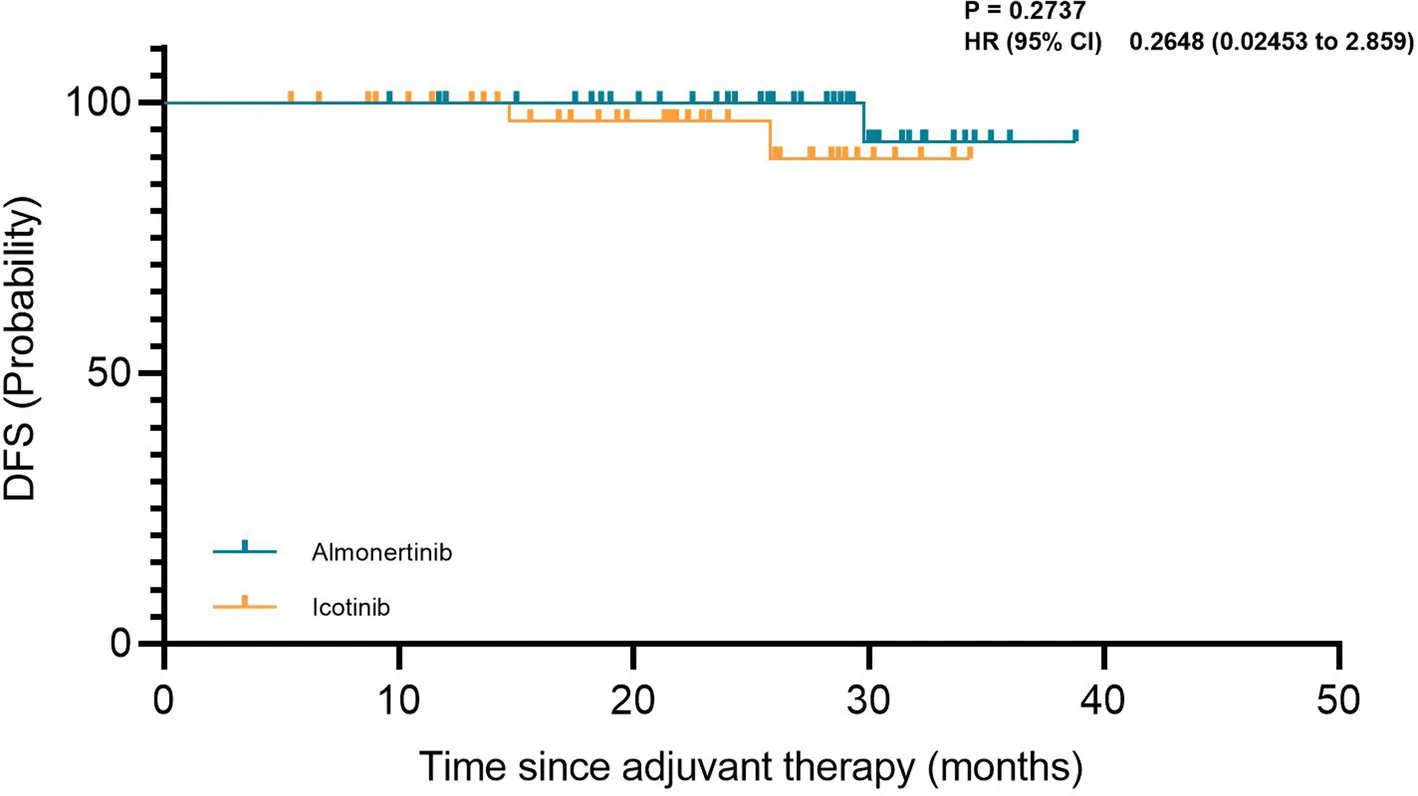
Kaplan–Meier curves of CNS metastasis-free survival. Only 3 CNS metastatic events were observed in the entire cohort; this CNS analysis is exploratory and cannot support definitive conclusions on CNS protective efficacy.

Among the 3 patients with CNS metastasis, 1 was in the almonertinib group and 2 in the icotinib group. Given the extremely low total number of CNS events (*n* = 3), the intergroup difference in 24-month CNS metastasis-free survival is only an exploratory numerical observation and cannot be used to draw robust conclusions about CNS recurrence prevention. At 24 months, all patients in the almonertinib group and 96.6% in the icotinib group were free from CNS metastasis (Figure 8).

Safety data were available for 79 patients, with 40 in the almonertinib group and 39 in the icotinib group. The overall incidence of TEAEs was 92.5% in the almonertinib group and 94.9% in the icotinib group. Grade ≥3 TEAEs occurred in 7.5% of the almonertinib group and 25.6% of the icotinib group (*p* = 0.0367). There were no statistically significant differences between the two groups in the incidence of any-grade TRAEs (85.0% vs. 89.7%, *p* = 0.7370) or grade ≥3 TRAEs (5.0% vs. 15.4%, *p* = 0.1543), as shown in Table 4. No TEAEs leading to death or TRAEs leading to death were observed in either treatment group.

**Table 4:** Summary of adverse events in the two treatment groups.

Adverse events	Almonertinib (*n* = 40)	Icotinib (*n* = 39)
Any-grade TEAEs	37 (92.5%)	37 (94.9%)
Grade ≥3 TEAEs	3 (7.5%)	10 (25.6%)
TEAEs leading to treatment interruption	1 (2.50%)	5 (12.8%)
Any-grade TRAEs	34 (85.0%)	35 (89.7%)
Grade ≥3 TRAEs	2 (5.0%)	6 (15.4%)

The most common adverse events in the Almonertinib group were diarrhea (30.0%), elevated creatine phosphokinase (CPK) (22.5%), and cough (22.5%), while the most common adverse events in the Icotinib group were decreased appetite (35.9%), neutropenia (33.3%), and Leukopenia (30.8%) (Table 5).

**Table 5:** Common treatment-related adverse events.

Adverse event	Almonertinib (*n* = 40)		Icotinib (*n* = 39)	
	Any grade	≥Grade 3	Any grade	≥Grade 3
Elevated CPK	9 (22.5%)	1 (2.5%)	1 (2.6%)	0
Elevated ALT	8 (20.0%)	1 (2.5%)	2 (5.1%)	0
Elevated AST	4 (10.0%)	0	1 (2.6%)	0
Thrombocytopenia	1 (2.50%)	0	10 (25.6%)	1 (2.6%)
Neutropenia	1 (2.50%)	0	13 (33.3%)	2 (5.1%)
Leukopenia	6 (15.0%)	0	12 (30.8%)	2 (5.1%)
Elevated bilirubin	2 (5.00%)	0	0	0
Decreased appetite	7 (17.5%)	0	14 (35.9%)	0
Vomiting	3 (7.50%)	0	11 (28.2%)	2 (5.1%)
Nausea	4 (10.0%)	0	9 (23.1%)	2 (5.1%)
Diarrhea	12 (30.0%)	1 (2.5%)	8 (20.5%)	1 (2.6%)
Anemia	7 (17.5%)	0	11 (28.2%)	1 (2.6%)
Cough	9 (22.5%)	0	6 (15.4%)	0
Pneumonia	4 (10.0%)	0	3 (7.7%)	0
Pruritus	5 (12.5%)	1 (2.5%)	4 (10.3%)	0
Rash	6 (15.0%)	0	5 (12.8%)	0
Myelosuppression	0	0	10 (25.6%)	0
Myalgia	2 (5.00%)	0	7 (17.9%)	0
Upper respiratory tract infection	2 (5.00%)	1 (2.5%)	0	0

## Discussion

Unlike patients with stage I NSCLC, who often achieve long-term cure with surgery alone, those with stage II-III disease continue to face substantial risks of postoperative recurrence and distant metastasis (13). Previous studies have shown that the addition of first-generation EGFR-TKIs such as icotinib after radical resection improves 5-year survival by only about 5% (14), while being accompanied by notable adverse effects and a high incidence of metastatic relapse (15).

Asian patients with lung adenocarcinoma have an EGFR mutation prevalence of approximately 50% (16), and EGFR-TKIs have demonstrated meaningful improvements not only in advanced disease but also in the adjuvant setting by significantly prolonging DFS and reducing both recurrence and distant metastasis in resected EGFR-mutant NSCLC (17–19). This real-world study compared the efficacy and safety of the third-generation EGFR-TKI almonertinib with the first-generation agent icotinib as adjuvant therapy in patients with completely resected stage II-IIIA EGFR-mutant lung adenocarcinoma. The results demonstrated that almonertinib was associated with significantly improved DFS, OS, and superior recurrence prevention compared with icotinib, while maintaining a favorable safety profile.

International guidelines increasingly recognize the pivotal role of osimertinib as adjuvant therapy for completely resected EGFR-mutant NSCLC. Based on the robust evidence generated by the phase III ADAURA trial, the European Society for Medical Oncology (ESMO) expert consensus recommends considering 3 years of adjuvant Osimertinib for patients with resected stage IB-IIIA EGFR-mutated disease (20). In ADAURA trial, Osimertinib established a new benchmark in the adjuvant setting, producing a profound improvement in DFS, with median DFS of 65.8 months versus 28.1 months (*p* < 0.001), and markedly higher 2-year and 4-year DFS rates (90% vs. 46 and 73% vs. 38%, respectively). These findings firmly support osimertinib as the preferred adjuvant therapy for patients with completely resected stage II-III EGFR-mutant NSCLC (1). Notably, these DFS gains translated into a significant overall survival benefit, with a 5-year OS of 88% versus 78% (*p* < 0.001) (21), confirming that third-generation EGFR-TKIs can meaningfully alter the long-term prognosis of early-stage EGFR-mutant NSCLC.

In 2025, results from the ARTS trial presented at the AACR annual meeting further demonstrated that almonertinib significantly improved mDFS and 2-year DFS compared with placebo in patients with resected stage II-IIIB EGFR-mutant NSCLC (mDFS: NR vs. 19.4 months; *p* < 0.001; 2-year DFS: 88.2% vs. 40.6%; *p* < 0.001) (22). These findings are consistent with our real-world analysis, which similarly showed superior mDFS and 2-year DFS with almonertinib versus icotinib (NR vs. 29.0 months; *p* = 0.0142; 85.1% vs. 61.2%). Differences in 2-year DFS rates between ARTS and our study, particularly in stage II and IIIA subgroups, may be attributable to variations in study design, sample size, and population heterogeneity. Notably, ARTS is a prospective placebo-controlled phase III trial with rigid trial-specified perioperative management and unified follow-up schedules, while our dual-center retrospective cohort lacks standardized trial constraints and contains more heterogeneous real-world patients. ARTS adopted a placebo comparator rather than icotinib, and there remains no published randomized trial directly comparing adjuvant almonertinib and icotinib; the numerical gap in DFS endpoints can only be explained through indirect cross-trial comparison rather than direct head-to-head inference.

Indirect comparisons also suggest that the mDFS observed in ADAURA (65.8 months) is longer than that in our cohort (NR at cutoff but unlikely to exceed the ADAURA estimate) (1), which could relate to differences in disease stage composition, sample size, and follow-up duration. Critical distinctions in treatment strategy and trial design further account for this survival gap: ADAURA allowed prior platinum adjuvant chemotherapy and included low-risk stage IB patients, relying on blinded prospective trial design with centralized follow-up. Our study excluded stage I cases, prohibited prior adjuvant chemotherapy, and was limited to retrospective real-world data with inevitable selection bias, creating non-negligible baseline and therapeutic differences between the two research populations. Moreover, approximately 61% of ADAURA participants received postoperative adjuvant chemotherapy before randomization, which may have contributed to the extended mDFS, whereas patients in the almonertinib group in our study did not receive prior adjuvant EGFR-TKIs. Similarly, earlier studies such as CTONG1104, IMPACT, and others generally reported higher 2-year DFS rates (17, 18, 23), likely due to differences in enrolled disease stages, patient selection, and treatment sequences. Despite these differences, the overall trend across clinical trials and real-world cohorts consistently supports the robust efficacy of third-generation EGFR-TKIs in the adjuvant setting. Conversely, earlier trials such as RADIANT and analyses by Kelly et al. (24) suggested limited benefit from adjuvant EGFR-TKIs, likely because treatment populations were not restricted to sensitizing EGFR mutations.

Although overall survival data in the present study remain immature, an early and consistent trend toward improved OS with almonertinib was observed. This benefit is consistent with the dual mechanism of third-generation EGFR-TKIs: potent suppression of sensitizing EGFR 19Del/L858R and prevention of T790M-mediated resistance (25), thereby delaying acquired resistance, together with enhanced blood–brain-barrier penetration that reduces CNS relapse. In our cohort, 24-month CNS metastasis-free survival was 100% with almonertinib versus 96.6% with icotinib, mirroring the CNS protective effect of osimertinib in ADAURA. This finding is directionally consistent with the ADAURA trial (1), which established a clear OS benefit for adjuvant third-generation EGFR-TKI therapy and confirmed that substantial DFS gains can translate into long-term survival improvement. Notably, in this research, the OS advantage of almonertinib appeared more pronounced in patients harboring EGFR 19Del. Similar stage and mutation-dependent patterns have been reported in ADAURA and earlier adjuvant EGFR-TKI trials, likely reflecting differences in baseline prognosis, tumor biology, and the impact of effective post-recurrence therapies. Taken together, these findings suggest that adjuvant almonertinib may offer meaningful survival benefit in selected patient subgroups, although longer follow-up is required to confirm the durability of this effect.

In addition to its efficacy advantages, almonertinib demonstrated a more favorable tolerability profile in the adjuvant setting. Grade ≥3 TEAEs occurred in only 7.5% of almonertinib-treated patients versus 25.6% in the icotinib group (*p* = 0.0367). Although the overall incidence of TEAEs was high and comparable between groups-consistent with the long treatment duration typical of adjuvant EGFR-TKI therapy-the incidence of grade ≥3 TEAEs was significantly lower in the almonertinib group than in the icotinib group. The most common almonertinib-related events were diarrhea, CPK elevation, and cough, virtually all grade 1–2 and easily managed; in contrast, icotinib was associated with a higher frequency of appetite loss, neutropenia, and leukopenia, reflecting its known myelosuppressive and gastrointestinal toxicity profile (26). Given that adjuvant EGFR-TKI therapy is administered for prolonged periods-often up to 2–3 years (27)-tolerability becomes a critical determinant of sustained treatment exposure and real-world effectiveness. The lower incidence of severe TEAEs observed with almonertinib may translate into fewer dose interruptions, reduced treatment discontinuation, and improved quality of life, thereby indirectly contributing to improved survival outcomes.

This study has several limitations. First, data were collected from only two medical centers and stage I patients were excluded, which may affect generalizability. In addition, follow-up time was inadequate to yield mature overall survival (OS) data. The total cohort size of 80 patients was relatively small, reducing statistical power and compromising the credibility of exploratory subgroup analyses with few clinical events. As a retrospective dual-center observational study without prospective design, no *a priori* sample size calculation was performed; all eligible patients receiving adjuvant almonertinib or icotinib within the study window were enrolled consecutively. The limited sample size largely stems from the relatively short clinical application history of adjuvant almonertinib and the restricted number of postoperative patients satisfying unified entry criteria at the two participating hospitals. All subgroup analyses in this work are exploratory rather than conclusive, given limited sample and event counts in each stratum. While the observed DFS and OS event counts satisfied regression modeling criteria to prevent overfitting, OS data remain immature with few deaths; any apparent OS benefit can only be interpreted as an early exploratory trend rather than a definitive conclusion.

Several research gaps remain to be addressed in future work. Subsequent studies could explore combined or sequential TKI regimens to identify patient subgroups with greater therapeutic gains. Previous adjuvant TKI trials demonstrated prolonged recurrence-free survival with 3-year versus 2-year treatment, warranting prospective trials comparing 2-year and 3-year almonertinib adjuvant therapy. Further dedicated research is needed to assess adjuvant almonertinib efficacy in special populations, such as patients with compound EGFR mutations, rare exon 20 insertions, or severe comorbidities. Finally, this analysis lacked detailed data on post-recurrence therapies, subsequent long-term survival, and CNS-specific time-to-event endpoints, which we plan to investigate in follow-up research.

## Conclusion

In summary, compared with icotinib, almonertinib demonstrated significantly superior efficacy and favorable tolerability as adjuvant therapy for patients with completely resected stage II-IIIA EGFR-mutant lung adenocarcinoma. Although an early OS trend favoring almonertinib was detected, the immature OS data preclude definitive conclusions on overall survival superiority. It offers a pragmatic genotype directed adjuvant option that can widen access across disparate health care systems, especially in cost constrained regions. These complementary real world data, aligned with emerging phase three evidence, establish almonertinib as an effective and affordable adjuvant therapy for resected EGFR-mutant NSCLC.

## Data Availability

The raw data supporting the conclusions of this article will be made available by the authors, without undue reservation.
